# The update of prostaglandin E2 receptor subtype 4 (EP4) in inflammatory diseases

**DOI:** 10.1016/j.gendis.2026.102169

**Published:** 2026-03-27

**Authors:** Shuxuan Li, Tong Wang, Anran Niu, Yuyan Du, Dong Zhou, Liang Gao, Haining Zhou, Shengnan Du

**Affiliations:** a Department of Pharmacology, School of Basic Medical Sciences, Zhengzhou University, Zhengzhou, Henan 450001, China; b The First Clinical School of Medicine, Zhengzhou University, Zhengzhou, Henan 450001, China; c Department of Gastroenterology, The First Affiliated Hospital of Zhengzhou University, Zhengzhou, Henan 450052, China; d The Third Clinical School of Medicine, Zhengzhou University, Zhengzhou, Henan 450001, China

**Keywords:** EP4, Inflammation, Inflammatory disease, PGE_2_, Signaling pathway

## Abstract

Inflammation is a defensive response of the organism to stimuli and is crucial for maintaining the stability of the internal environment. However, abnormal inflammatory responses, including over-activation or hypo-responsiveness, are among the major factors causing numerous diseases. Among all prostaglandin substances, PGE_2_ is one of the common lipid mediators produced by the catalysis of arachidonic acid (AA) by cyclooxygenase (COX). Through autocrine or paracrine secretion, PGE_2_ acts on four receptor subtypes (*i.e.*, EP1, EP2, EP3, and EP4) and participates in various physiological and pathophysiological processes, commonly including fever, pain, and inflammation. Among them, EP4 is deeply researched and mediates many effects of PGE_2_, such as fever, pain, inflammation, blood pressure regulation, water-salt metabolism, and tumorigenesis. In particular, the mechanism of action and potential therapeutic value of EP4 in inflammatory diseases have been research hotspots for years. Here, we summarize the function of the PGE_2_–EP4 signaling in inflammation and related inflammatory diseases.

## Introduction

Inflammation, as one of the indispensable defense mechanisms of the immune system, has the core function of rapidly activating the immune response when the body is exposed to external pathogens or noxious stimuli, aiming at removing inflammation-causing factors, thus maintaining the homeostasis of the internal environment of the body. However, when the inflammatory response deviates from its normal track and manifests itself as over-activated, under-activated, or abnormally regulated, it becomes a central driver of numerous pathological states. Examples of such inflammatory diseases include arthritis, ulcerative colitis, viral hepatitis, *etc*. A variety of inflammatory mediators, such as cytokines, chemokines, and prostaglandins, play key roles in the inflammatory process.^1^ They are involved in the initiation, development, and abatement of inflammatory responses through a complex network of regulatory mechanisms. Among them, prostaglandins transmit inflammatory signals and regulate multiple physiological and pathological processes, such as vascular permeability and leukocyte migration, as well as pain perception, by binding to their receptors.^2^ The role of prostaglandin E_2_ (PGE_2_), a major prostaglandin, exemplifies this complexity. PGE_2_ is crucial in inflammation, and non-steroidal anti-inflammatory drugs (NSAIDs), which inhibit its synthesis, are widely used in clinical anti-inflammatory therapy. However, the clinical application of NSAIDs is ineffective in certain inflammatory diseases (such as ulcerative colitis) and may even exacerbate their clinical symptoms.^3^ This paradoxical effect may be attributed to the dual roles of PGE_2_ as both a pro-inflammatory and an anti-inflammatory mediator. These opposing effects typically participate in different stages of various inflammations in a context-dependent manner through PGE_2_'s interactions with its four distinct receptors.

## Overview of the EP4 signaling pathway

Prostaglandins are a class of twenty-carbon unsaturated fatty acids synthesized through the metabolism of arachidonic acid. Among them, PGE_2_ is the most extensively studied prostaglandin. As an endogenous ligand, PGE_2_ has four receptor subtypes, namely EP1, EP2, EP3, and EP4. Among these subtypes, EP4 has the broadest distribution (including the gastrointestinal tract, cardiovascular system, kidneys, lungs, hematopoietic tissues, skin, *etc*.) and is significantly involved in inflammatory disease, cardiovascular disease, renal disease, and cancer.^4^^,^^5^

The diverse biological functions of EP4 are closely related to its diverse signaling pathways. Firstly, as a G-protein-coupled receptor (GPCR), EP4 has seven transmembrane domains and can couple with different G-protein subtypes (Gαs and Gαi) upon activation to transduce various transmembrane signals. The classic activation pathway of EP4 is coupled with the stimulatory G-protein (Gαs). Under the stimulation of the endogenous ligand PGE_2_ or agonists, it activates adenylate cyclase (AC), increasing intracellular cyclic adenosine monophosphate (cAMP) levels. cAMP further activates protein kinase A (PKA) or exchange protein activated by cAMP (Epac). PKA phosphorylates downstream proteins to regulate physiological and pathophysiological processes. For example, in the cardiovascular system, the activation of EP4 in endothelial cells induces vasodilation or promotes angiogenesis through the cAMP–PKA pathway.^6^^,^^7^ In addition, PKA can directly activate kinases such as glycogen synthase kinase-3 (GSK3), protein kinase B (AKT), adenosine monophosphate-activated protein kinase (AMPK), and mitogen-activated protein kinases (MAPKs), exerting physiological or pathological effects.^3^^,^^4^^,^^8^^,^^9^ Beyond the direct effects of PKA outlined above, this kinase can also activate the nuclear transcription factor cAMP response element-binding protein (CREB). For instance, in bone marrow cells, PGE_2_ binds to EP4 and triggers the cAMP–PKA pathway, which in turn activates CREB to promote cell differentiation.^10^ In monocytes, EP4-mediated CREB activation up-regulates the transcription and translation of chemokine receptor 7 (CCR7), thereby regulating monocyte chemotaxis.^11^ Within the kidney, the activation of the EP4–cAMP–PKA pathway in collecting duct epithelial cells increases the urine reabsorption by up-regulating the aquaporin 2 (AQP2) expression via CREB.^12^ In osteoarthritis, the EP4 receptor on articular chondrocytes modulates cartilage anabolism and catabolism via the cAMP–PKA–CREB–Sox9 signaling axis, thereby influencing cartilage regeneration and post-injury joint pain.^13^ Furthermore, the Epac signaling pathway, another cAMP effector that acts independently of PKA, is also important in the cardiovascular and musculoskeletal system. For example, Epac1 in cerebral microvascular endothelial cells mediates PGE_2_/EP4-induced ICAM1 expression, while Epac1 in arterial duct smooth muscle cells affects PGE_2_/EP4-mediated cell migration, further influencing the arterial duct closure.^14^^,^^15^ The presence of the PGE_2–_EP4–cAMP–Epac1–Rap1 axis in rheumatoid synovial fibroblasts is closely related to rheumatoid arthritis.^16^ Since both EP4 and EP2 are coupled with Gαs-protein and activate cAMP-mediated signaling pathways, they have similar and compensatory roles in cells or disease. Multiple studies have confirmed the inhibitory effects of PGE_2_–EP2/EP4–cAMP signaling in cell migration, degranulation, chemotaxis, adhesion, phagocytosis, inflammatory mediator synthesis in immune cells, including eosinophils, neutrophils, macrophages, dendritic cells, and natural killer cells.^17^ Especially in the tumor microenvironment, both EP2 and EP4 in tumor immune cells are jointly activated by the high concentration of PGE_2_, transmit signals via the Gαs–cAMP–PKA pathway, and exhibit both functional redundancy and synergistic complementarity in mediating immune escape.^18^^,^^19^ Nonetheless, there are still some disparities between them. For example, PGE_2_ exerts bronchodilatory and anti-inflammatory effects in the lung via EP2 and EP4; however, EP2 is more potent in anti-inflammatory effects, whereas EP4 primarily mediates bronchodilation. The discrepancy between EP2 and EP4 observed here may be that the lung inflammatory immune cells responsible for regulating inflammation highly express EP2, whereas airway smooth muscle cells that affect airway contraction highly express EP4.^20^ Another interpretation for this discrepancy may be that the cAMP-elevating response of EP4 to PGE_2_ is weaker than that of EP2.^21^ This result may stem from the additional inhibitory G-protein (Gαi) coupling found in EP4, but not in EP2, which has opened the discussion about the possibility that EP4 receptors function in multiple signaling pathways and have biased activities. In detail, EP4 couples with Gαi, which inhibits AC activity upon PGE_2_ stimulation, leading to a decrease in cAMP levels. In 2023, the cryo-EM structures of EP4–Gαs and EP4–Gαi complexes bound to the endogenous ligand PGE_2_ were determined, and the signaling bias of multiple EP4 agonists was mapped. Within this framework, Gαs-biased EP4 signaling has been shown to underlie the receptor's protective roles in drug-induced renal injury and inherited diabetes insipidus.^22^ Although both EP3 and EP4 can couple with Gαi proteins to inhibit cAMP synthesis, they mediate divergent outcomes in physiological and pathophysiological processes. In detail, EP3–Gαi signaling principally mediates vasoconstriction, inhibition of gastric acid secretion, and adipogenesis under physiological conditions.^23^, ^24^, ^25^ Under pathological conditions, persistent activation of EP3–Gαi signaling leads to chronic suppression of cAMP, thereby contributing to the pathogenesis of multiple diseases: hypertension, thrombosis, and atherosclerosis in the cardiovascular system; obesity, insulin resistance, and fatty liver disease in the metabolic domain; and accelerating cancer progression in tumors by inhibiting apoptosis while promoting proliferation, angiogenesis, metastasis, and immune escape. However, the detailed role of the EP4–Gαi signaling pathway remains unknown, and further work is needed in this area.

Beyond their G protein-coupling effects, multiple studies have confirmed that the EP4 receptor exhibits biased activation pathways. For instance, its intracellular domain can activate distinct signaling cascades by interacting with proteins such as EP4 receptor-associated protein (EPRAP) and β-arrestin. EPRAP, first identified by Takayama et al in macrophages, mediates the anti-inflammatory effects of EP4. EP4 is related to various inflammatory diseases, such as atherosclerosis, enteritis, and pneumonia.^26^, ^27^, ^28^ The specific anti-inflammatory mechanism involves stabilizing the p105 subunit of nuclear factor kappa B (NF-κB), thereby inhibiting the activation of NF-κB and extracellular signal-regulated kinase 1/2 (ERK1/2) signaling, subsequently reducing the pro-inflammatory cytokines.^29^ Furthermore, the C-terminus of the EP4 receptor can be phosphorylated by G-protein-coupled receptor kinase 2/3/5 (GRK2/3/5).^30^ Phosphorylated EP4 can also bind to β-arrestin1.^31^ In colorectal cancer, phosphorylation-dependent binding of EP4 to β-arrestin1 trans-activates cellular-Src, leading to activation of the epidermal growth factor receptor (EGFR) and phosphoinositide 3-kinase (PI3K)–Akt pathway; conversely, in the intestinal mucosa, EP4 activates the AKT signaling pathway via β-arrestin1 to alleviate mucosal damage.^32^ Moreover, in osteoarthritis, the PI3K–AKT–MAPK signaling pathway can diminish subchondral bone formation and inhibit sensory-inervation density, thereby alleviating osteoarthritis progression and pain.^33^ In dextran sulfate sodium-induced chronic intestinal inflammation, EP4 activation in macrophages engages the MAPK pathway, thereby enhancing the synthesis and secretion of C-X-C motif chemokine ligand 1 (CXCL1) and promoting mucosal repair.^34^ Besides conducting EP4 signals, GRK2 and β-arrestin are also involved in EP4 receptor desensitization. In detail, EP4 activation undergoes internalization and degradation through coupling with GRK2 or β-arrestin2/3.^35^, ^36^, ^37^ EP4 desensitization results in lower cAMP levels, which helps explain why EP4's cAMP-raising response to PGE_2_ is less pronounced than that of EP2.

**Figure 1:**
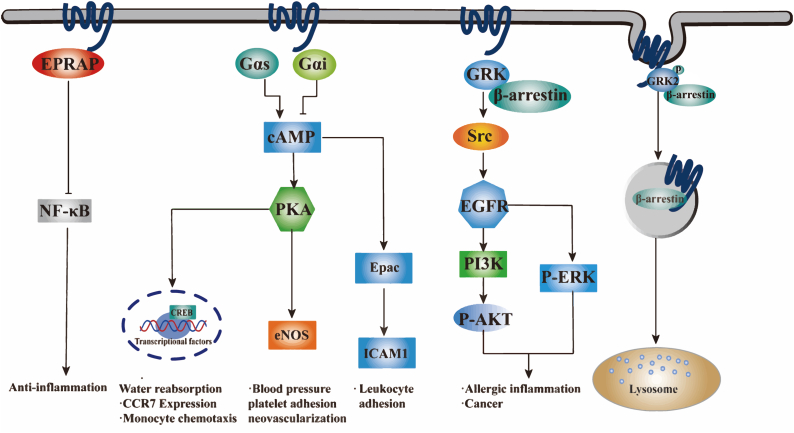
The structure of the EP4 receptor and its signaling pathways. EP4, a G protein-coupled receptor (GPCR), binds to prostaglandin E2 (PGE_2_) and activates Gαs, increasing cAMP and PKA activity. EP4 also couples with Gαi, inhibiting adenylate cyclase (AC) and counteracting Gαs effects. Additionally, EP4 engages NF-κB, PI3K/Akt, and β-arrestin pathways, regulating inflammation, cell proliferation, and tissue repair. Widely expressed in immune, cardiovascular, and reproductive systems, EP4 plays critical roles in physiological and pathological processes.

In conclusion, EP4 receptor activation initiates at least four distinct signaling pathways. The classical pathway involves the dissociation of Gαs subunits and Gβγ dimers, with Gαs stimulating the synthesis of cAMP through the activation of AC. This, in turn, activates downstream effectors such as Epac (a cAMP-activated exchange factor) and PKA, ultimately enhancing the phosphorylation of endothelial nitric oxide synthase (eNOS) and the transcription factor CREB. However, EP4 activation can also lead to a reduction in cAMP levels via the activation of Gαi proteins. Additionally, as a GPCR, the EP4 receptor undergoes rapid desensitization when GRK phosphorylates serine residues at its C-terminus. This phosphorylation of EP4 can attract β-arrestin, facilitating receptor internalization or triggering the activation of EGFR. Furthermore, the extended C-terminus of the EP4 receptor can interact with EPRAP, thereby initiating anti-inflammatory responses (Fig. 1).

## The role of EP4 in inflammatory disease

**Figure 2:**
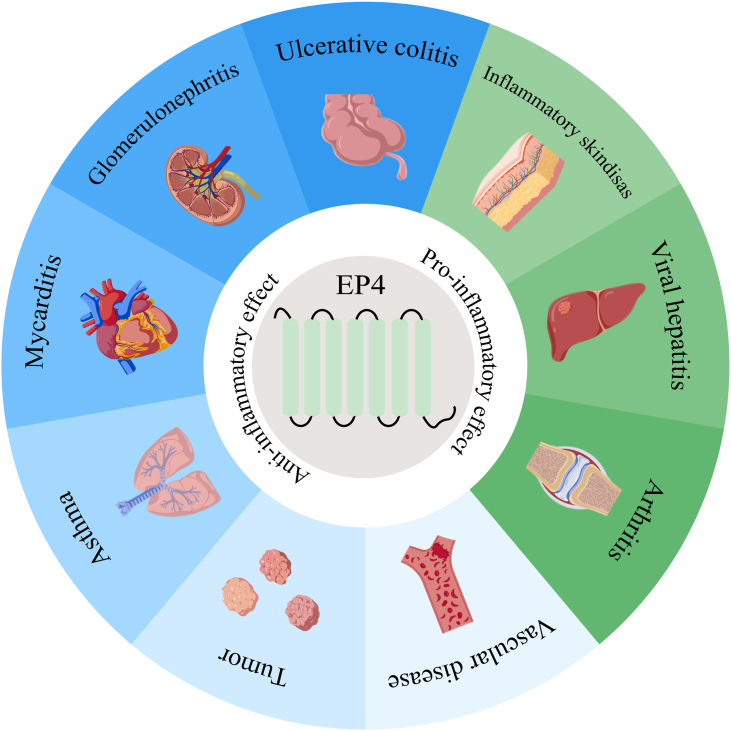
The relationship between the four subtypes of prostaglandin E2 receptors and inflammatory diseases. Arthritis, viral hepatitis, and inflammatory skin diseases are associated with pro-inflammatory effects, while asthma, tumor, vascular disease, myocarditis, glomerulonephritis, and ulcerative colitis are associated with anti-inflammatory effects.

PGE_2_ is important in inflammation, and NSAIDs, which inhibit its synthesis, are widely used in clinical anti-inflammatory therapy. However, the clinical application of NSAIDs is ineffective in certain inflammatory diseases (such as ulcerative colitis) and may even exacerbate their clinical symptoms.^3^ This may be due to the dual roles of PGE_2_ as both a pro-inflammatory and an anti-inflammatory mediator, which typically participate in different stages of various inflammations in a condition-dependent manner through four receptors. Among them, EP4 is involved in the majority of PGE_2_-mediated pro-inflammatory and anti-inflammatory processes. EP4 has been reported to be associated with multiple inflammatory diseases, including locomotor system inflammations represented by rheumatoid arthritis, osteoarthritis, and ankylosing spondylitis; digestive system inflammations represented by inflammatory bowel disease (IBD); urinary system inflammations represented by glomerulonephritis; and autoimmune diseases such as multiple sclerosis and atopic dermatitis. The details are elaborated below (Fig. 2).

## Arthritis

### Rheumatoid arthritis

Rheumatoid arthritis is a complex and prevalent systemic autoimmune inflammatory disease characterized by abnormal synovial hyperplasia and progressive destruction of the joints.^38^ Currently, the primary clinical therapeutic drugs for this disease are PGE_2_ synthesis inhibitors. Compelling evidence shows that increased PGE_2_ synthesis in the blood and synovial tissues of rheumatoid arthritis patients promotes the occurrence and progression of rheumatoid arthritis.^39^ Moreover, using systemic gene knockout mice for the four PGE_2_ receptors, EP1, EP2, EP3, and EP4, demonstrated that deficiency of EP4 alone inhibited the symptoms and pathological damage of collagen antibody-induced arthritis, and significantly suppressed serum levels of IL-6 and serum amyloid A.^40^ However, deficiency of EP1, EP2, or EP3 alone left arthritis severity unchanged, whereas administration of the EP4 antagonist ONO-AE3-208 markedly attenuated disease in EP2-deficient mice. This indicates functional redundancy between EP2 and EP4 in collagen-induced arthritis, likely due to their overlapping downstream signaling pathways.^41^ Consistently, other EP4 antagonists (*e.g.*, ER-819762, CJ-042794, and CJ-023423) have demonstrated efficacy in inhibiting both collagen-induced arthritis and adjuvant-induced arthritis.^42^^,^^43^ Pharmacological inhibition of EP4 is therapeutic.^37^ For example, paeoniflorin-6′-O-benzenesulfonate (CP-25) could inhibit the activation of the PGE_2_–EP4 signaling and indirectly restrain aberrant fibroblast-like synoviocyte proliferation, regulate dendritic cell effector functions, and redirect macrophage polarization, collectively ameliorating the multi-level pathogenesis of rheumatoid arthritis.^44^^,^^45^ In summary, the PGE_2_–EP4–cAMP axis is a central pathogenic hub in rheumatoid arthritis, driving synovitis, immune dysregulation, and joint destruction. Genetic and pharmacological evidence confirms that targeted EP4 inhibition effectively suppresses arthritis across models by normalizing dendritic cell function, curbing FLS proliferation, and modulating macrophage responses. This dual-pathology mechanism—where aberrant EP4 activation fuels disease while its blockade confers protection—positions EP4 antagonists as next-generation disease-modifying therapies beyond symptomatic NSAIDs.

### Osteoarthritis

Osteoarthritis is not merely a mechanical degeneration of cartilage, but also affects the entire joint system, with cartilage, subchondral bone, and synovium all potentially playing key roles in the pathogenesis of the disease. Furthermore, osteoarthritis is associated with systemic inflammation.^46^ Recently, EP4 has emerged as a promising therapeutic target for osteoarthritis.^33^ However, PGE_2_–EP4 plays a dual role in the pathological process of osteoarthritis. First, chondrocytes obtained from patients with advanced osteoarthritis revealed that PGE_2_ inhibited proteoglycan synthesis and promoted matrix degradation via the EP4 in osteoarthritis chondrocytes.^47^ Moreover, several antagonists of EP4 (grapiprant, ER-819762, AH23848, and HL-43) have been shown to alleviate joint inflammation and pain symptoms in animal models of osteoarthritis, which involves EP4 regulation of osteoblast differentiation, inflammatory cell proliferation and activation, subchondral bone angiogenesis, and cartilage synthesis.^13^^,^^33^^,^^48^ Correspondingly, activation of EP4 in articular chondrocytes can inhibit the expression of matrix metalloproteinases, suppress the growth of osteophytes, and show its arthroprotective effects.^49^^,^^50^ The nature of EP4 receptor signaling in osteoarthritis—whether it exerts pro-inflammatory or protective effects—may depend on the target cell type, disease progression stage, activated downstream signaling pathways, and the surrounding receptor signaling microenvironment. Thus, the specific role and mechanism of EP4 in osteoarthritis still need to be further explored.

### Ulcerative colitis

Prolonged, high-dose NSAIDs in clinical use can cause gastrointestinal mucosal damage, exacerbate inflammation, and potentially induce or exacerbate IBD, primarily by inhibiting the synthesis of metabolites like PGI_2_ and PGE_2_.^51^^,^^52^ PGE_2_ exerts its pathophysiological function mainly through EP4. EP4 is encoded by the PTGER4 gene, whose mutations can alter EP4 expression and are closely associated with IBD susceptibility. Currently, several PTGER4 polymorphic loci have been reported, such as rs4495224, rs7720838, rs17234657, and rs4613763, which are associated with the development of ulcerative colitis or Crohn's disease.^53^^,^^54^ Furthermore, EP4 is always highly expressed in intestinal epithelial cells in IBD patients or animal models.^55^ Various EP4 agonists (such as AGN205203, KAG-308, ONO-4819, ONO-AE1-734, and NXT-10796) have been demonstrated to promote intestinal mucosal repair and to ameliorate the symptoms of IBD and intestinal injury by inhibiting intestinal epithelial cell necrosis, decreasing the infiltration of immune cells, and promoting lymphangiogenesis.^56^, ^57^, ^58^, ^59^, ^60^ In addition, some comparative studies have found that EP4 agonists are more effective in IBD treatment than the commonly used drugs for IBD, such as aminosalicylates (sulfasalazine, mesalazine, olsalazine), corticosteroids (budesonide), 6-thioguanine, cyclosporine A (CsA), *etc*.^61^ Furthermore, a phase II clinical trial has confirmed that the EP4 agonist ONO-4819 is effective in IBD patients resistant to 5-aminosalicylates.^62^ Consistent with the results of the agonists mentioned above, studies using EP4 transgenic animals have also validated the protective role of EP4 in IBD.^34^^,^^59^ In detail, among various prostaglandin receptors (DP, EP1, EP2, EP3, FP, IP, and TP), only globally EP4-deficient mice exhibited significant impairment of mucosal barrier function, epithelial loss, crypt damage, and aggregation of neutrophils and lymphocytes in the colon compared with control mice.^59^ Moreover, deficiency of EP4 specifically in macrophages exacerbated the prolonged inflammatory injury in the IBD model, due to the inefficient repair of the intestinal mucosa.^34^ PGE_2_–EP4 signaling acts directly on group 3 innate lymphoid cells, promoting their homeostasis and driving them to produce interleukin-22 (IL-22), which is essential in protecting against gut barrier dysfunction.^63^ However, there are still some studies that are inconsistent with the above results. During IBD, human intestinal fibroblasts transform into inflammation-associated fibroblasts, which synthesize and secrete PGE_2_, acting on EP4 of intestinal epithelial cells to promote edema and barrier damage of the intestine.^64^ Additionally, loss of EP4 in CD4^+^ T cells reduces the infiltration of CD4^+^FoxP3^+^ and CD4^+^RORγt^+^ cells in mesenteric lymph nodes and colonic tissues, and finally attenuates IBD.^65^ Furthermore, in a T cell-initiated age-related intestinal inflammation mouse model, pharmacologically blocking EP4 or genetically ablating EP4 in mononuclear phagocytes diminishes age-related impairment of intestinal inflammation resolution through fostering gut microbial dysbiosis and, more importantly, interrupting segmented filamentous bacterial adhesion to the intestinal epithelium.^66^ The seemingly contradictory roles of the EP4 receptor in IBD can be dissected along several key dimensions. First, EP4 exerts opposed effects in distinct cell types, and these cell types selectively engage different downstream effectors of PGE_2_/EP4 signaling. Second, variations in the experimental models employed—together with differences in the microenvironmental milieu, including the gut microbiota and its metabolites—can collectively yield divergent outcomes regarding EP4 function. Thus, more in-depth mechanistic studies are needed to elucidate the role of EP4 in IBD and to provide the theoretical basis for the development of IBD therapeutic drugs targeting this receptor.

### Viral hepatitis

Viral hepatitis is the primary stage of cirrhosis and hepatocellular carcinoma. Common chronic hepatitis is caused by infections with the hepatitis B virus (HBV) and the hepatitis C virus (HCV). Ultimately, the immune system dysregulation after viral infection leads to liver dysfunction.^67^ Currently, studies have confirmed that the up-regulation of PGE_2_ and COX-2 occurs in patients with hepatitis B and hepatitis C, as well as in mouse models.^68^^,^^69^ Moreover, the level of serum PGE_2_ in patients with hepatitis B is positively correlated with the severity of liver injury. In detail, PGE_2_ engagement of EP4 activates the PI3K/Akt pathway, leading to phosphorylation and inactivation of GSK3β and Bad, thereby suppressing apoptosis in virus-infected hepatocytes.^68^ Furthermore, *in vitro*, lymphocytic choriomeningitis virus infection has been found to up-regulate EP2 and EP4 in virus-specific cytotoxic T lymphocytes, enabling PGE_2_ to suppress their survival and cytolytic activity.^70^ In summary, PGE_2_-EP4 plays a role by affecting the infectivity and replication efficiency of viruses in host cells or by regulating the host's immune response to viral infection. Therefore, EP4 may be a promising therapeutic target for hepatitis viruses.

### Glomerulonephritis

Glomerulonephritis is a complex renal inflammation that is always induced by autoimmune reactions, infectious diseases, malignant tumors, or metabolic disorders. The characteristic pathological features include glomerular crescent formation and leukocyte infiltration.^71^^,^^72^ Mouse models of glomerulonephritis exhibit significantly elevated levels of PGE_2_ in glomeruli. Moreover, EP4 agonist ONO-AE1-329 has been shown to suppress the formation of glomerular crescents and the deposition of IgG, effectively halting the progression of glomerulonephritis.^73^ Pharmacological inhibition of PGE_2_ production or activation of EP4 in adipose stromal cells could alleviate IgG-induced nephritis and renal injury.^74^ However, the EP4-selective antagonist ONO-AE3-208 alleviates the pathological changes of serum-induced nephritis by suppressing CXCL1 and CXCL5, thereby decreasing the infiltration of neutrophils into the renal interstitium.^75^ Likewise, EP4 antagonist AH23848 inhibits nitric oxide (NO) by decreasing cAMP in mesangial cells, and mitigating renal inflammation.^76^ The paradoxical effects of the EP4 receptor in glomerulonephritis may stem from two reasons: the bidirectional regulation of signaling pathways (EP4 activation exerts context-dependent opposing effects in different scenarios) and target cell specificity (the functional outcomes may diverge or even oppose each other depending on the distinct target cells involved). The controversial results of EP4 agonists and antagonists indicate that the mechanism of EP4 in glomerulonephritis should be investigated in depth.

### Myocarditis

Myocarditis is an immunoinflammatory heart disease, and is primarily induced by infections, such as viruses, bacteria, fungi, and protozoa. Among these, viral myocarditis is the most common cause of heart failure. The up-regulated expression of COX-2 and mPGES1, leading to increased PGE_2_ synthesis, is common in myocarditis patients or animal models.^77^ However, the exogenous administration of PGE_2_ markedly attenuates disease progression in the EAM model.^78^ Therefore, the therapeutic effects of NSAIDs in myocarditis-related clinical or animal experiments are still debated.^79^, ^80^, ^81^ However, EP4 agonists (EP4RAG and ONO-0260164) have been shown to improve cardiac function, attenuate myocardial fibrosis, suppress T-cell infiltration and proliferation, and down-regulate the pro-inflammatory cytokine monocyte chemoattractant protein-1 (MCP-1) in mice with myocarditis.^82^^,^^83^ The above findings imply the protective role and potential target of EP4 in myocarditis.

### Inflammation-related vascular disease

Atherosclerosis is a prevalent chronic vascular inflammatory disease characterized by lipid deposition and inflammatory responses in the vessels. Since the expression of EP4 in human atherosclerotic plaques positively correlates with the degree of plaque inflammation,^84^ studies using similar models have shown that marrow EP4-deficient Ldlr-knockout mice do not affect inflammation or the formation of early atherosclerotic plaques (5 weeks of high-fat diet). But at a later stage (10 weeks of high-fat diet), bone marrow EP4-deficient mice exhibited heightened plaque inflammation, accompanied by an increase of inflammatory cytokines (monocyte chemoattractant protein-1 and interferon-γ inducible protein 10), T-lymphocyte infiltration, and a greater risk of plaque rupture.^85^ In support of this, in an ApoE knockout atherosclerosis model, myeloid-specific EP4 deficiency enhances foam cell formation and M1 polarization by up-regulating CD36, thereby accelerating the progression of atherosclerosis.^86^ These findings support the role of EP4 as an anti-inflammatory mediator in atherosclerosis. This protective role may depend on the EPRAP, an EP4-binding protein, which inhibits macrophage activation by sequestering NF-κB.^29^^,^^87^ However, its role in diabetes-accelerated atherogenesis remains unclear, as macrophage EP4 deficiency does not alter this process despite enhanced PGE_2_–EP4 signaling in type 1 diabetes mellitus.^88^ Similar to atherosclerosis, an aortic aneurysm is another common inflammatory vascular disease. However, the role of EP4 in aneurysms remains highly controversial. On one hand, suppressing EP4 signaling—either through pharmacological antagonism or vascular smooth muscle cell-specific haploinsufficiency—has been shown to attenuate aortic aneurysm formation in apolipoprotein E-deficient mouse models.^89^^,^^90^ However, contrasting results showed that myeloid macrophage EP4 deficiency promoted the development of angiotensin II (Ang II)-induced abdominal aortic aneurysms.^91^ Similarly, research has demonstrated that vascular smooth muscle cell-specific EP4 deficiency exacerbates Ang II-induced vascular inflammation, worsening aortic dissection and aneurysms.^92^ The apparent contradiction in the role of EP4 in vascular inflammatory diseases likely stems from two core factors. First, the dominant cell types involved shift dynamically across disease stages, endowing EP4 with stage-specific biological effects. Second, EP4-mediated modulation of the inflammatory milieu appears to be temporally gated: its actions differ markedly between the acute and chronic phases. Therefore, further investigation is required to elucidate its specific mode of action.

### Inflammatory skin disease

Cutaneous inflammatory diseases arise from disruptions of the physical and immunological barriers, characterized by immune dysregulation that leads to diverse pathological conditions, including contact dermatitis, atopic dermatitis, and psoriasis. A shared pathological hallmark of these disorders is T cell-mediated or antigen-antibody-mediated inflammation, in which the PGE_2_–EP4 signaling pathway plays a key role.

Contact dermatitis and atopic dermatitis are prevalent inflammatory skin disorders characterized by dysfunctions of dendritic cells or T cells in their pathogenesis. The genes included in the PGE_2_ signaling pathway, including PGE_2_ synthases (COX-2), EP2, and EP4 receptors, are abundantly present in human psoriatic skin lesions.^93^ In pre-clinic studies, the mouse contact hypersensitivity (CHS) experimental model, induced by common haptens such as 2,4-dinitro-1-fluorobenzene (DNFB) and oxazolone, is widely used for these conditions. Studies demonstrated that in a DNFB-induced CHS model, both EP4-deficient mice and mice treated with the EP4 antagonist ONO-AE3-208 exhibited attenuated contact hypersensitivity responses. During the sensitization phase, these mice also displayed reduced trafficking of antigen-loaded skin dendritic cells to draining lymph nodes via CCL21-dependent chemotaxis. These findings collectively highlight the critical role of EP4 in mediating dendritic cell maturation and migration during CHS pathogenesis.^94^ Two distinct helper T (Th) subsets, Th1 and Th17, mediate tissue damage and inflammation in allergic skin disorders. Notably, Yao et al extended these observations *in vitro* and *in vivo*, revealing that PGE_2_ mediates tissue damage and inflammation in allergic skin disorders by regulating two distinct Th subsets, Th1 and Th17. In detail, PGE_2_ initiates Th1 cell differentiation via PI3K and facilitates IL-23-induced Th17 cell expansion via cAMP through EP2 and EP4. Additionally, PGE_2_–EP4 signaling indirectly promotes Th17 differentiation by enhancing IL-23 production from dendritic cells.^95^ Administration of an EP4-selective antagonist *in vivo* decreases the accumulation of both Th1 and Th17 cells in regional lymph nodes and suppresses disease progression in mice subjected to contact hypersensitivity.^95^ PGE_2_ mobilizes T cell receptor (TCR) signaling, such as (IL-12/IL-12R and IFN-g/IFN-gR) in T cells through the EP4-dependent cAMP–PKA and PI3K pathway. Loss of EP4 in T cells restricts expression of IL-12Rb2 and IFN-γR1, and attenuates Th1 cell-mediated contact hypersensitivity *in vivo*.^96^

Since a positive correlation between the mRNA expression levels of PGE_2_ synthases, EP2/EP4 receptors, and IL-22 in human atopic dermatitis lesions, experimental studies confirmed that PGE_2_ induced IL-22 secretion from T cells via EP2/EP4–cAMP signaling in an oxazolone-induced atopic dermatitis model. Selective deletion of EP4 in T cells, which prevents IL-22 production, limits atopic-like skin inflammation in this model.^93^ Additionally, further investigations revealed that EP4 deficiency in T cells increased regulatory T cell generation with a reduced number of effector T cells in the draining lymph nodes after sensitization with haptens. Mechanistically, PGE_2_–EP4 signaling interrupts transforming growth factor beta (TGF-β)-driven forkhead box P3 (Foxp3) induction during induced regulatory T cell differentiation.^97^

In contrast, PGE_2_ also exerts a protective effect in specific contexts of atopic dermatitis. Studies in a *Dermatophagoides farina-*induced murine atopic dermatitis model showed that PGE_2_ derived from human umbilical cord blood mesenchymal stem cells inhibited mast cell degranulation, reducing histamine release and the production of inflammatory factors such as TNF-α, thereby alleviating CHS. However, the EP receptors involved in this process were not mentioned.^98^ Current evidence suggests that EP3 may mediate the beneficial effects of PGE_2_ in CHS. Mechanistically, PGE_2_–EP3 signaling has been shown to suppress cutaneous dendritic cell migration to draining lymph nodes after sensitization in CHS. Interestingly, the inhibitory functions of PGE_2_–EP3 signaling on dendritic cell migration are observed only when the amount of antigen is suboptimal and not when the antigen dose is high.^99^ Additionally, PGE_2_–EP3 signaling also inhibits keratinocyte activation and exerts anti-inflammatory actions in murine contact hypersensitivity by inhibiting neutrophil-recruiting chemokines, including CXCL1, at the elicitation site.^100^

Human psoriatic skin biopsy specimens show a positive correlation between PGE_2_ signaling and the IL-23/Th17 pathway. Lee et al investigated that PGE_2_ supported the development of psoriatic dermatitis by promoting the IL-23 pathways in Th17 cells. IL-23 stimulates Th17 cells to produce PGE_2_, which acts back on EP2 and EP4 in these cells to further augment IL-23r expression in a positive feedback loop, ultimately accelerating the IL-23-induced expansion of Th17 cells in the skin of psoriatic dermatitis. Combined deletion of EP2 and EP4 selectively in T cells suppressed the accumulation of IL-17A^+^ and IL-17A^+^IFN-γ^+^ pathogenic Th17 cells and abolished skin inflammation in an IL-23-induced psoriasis mouse model.^101^ The above evidence indicates that EP4 plays a significant role in inflammatory skin disorders and that an EP4 antagonist could be a good therapeutic drug target for intractable dermatitis.

### Tumor

In 1863, the infiltration of inflammatory cells in tumors was discovered, establishing an early correlation between inflammation and tumors.^102^ To date, the complex relationship between inflammation and tumors has been progressively elucidated. For instance, anti-inflammatory drugs that inhibit the synthesis of PGE_2_, represented by NSAIDs such as aspirin, can reduce the incidence and mortality risk of cancer.^103^, ^104^, ^105^ To this day, in addition to its direct effects on tumor cells, researchers have paid more attention to the role of the EP4 signaling in tumor immunity. Specifically, PGE_2_ induces immunosuppression through EP4 in inflammatory cells of the tumor microenvironment.^19^ Firstly, PGE_2_ stimulates the differentiation and proliferation of macrophages, like tumor-associated macrophages, and myeloid-derived suppressor cells in tumors through EP4, promotes the expression of immune-suppressive factors such as PD-L1 and ARG-1, and suppresses the tumor immunity.^106^, ^107^, ^108^ Furthermore, the direct or indirect activation of PGE_2_/EP4 signaling in T cells induces the activity of regulatory T cells, inhibits the function of natural killer cells,^109^ suppresses the differentiation and maturation of cytotoxic T cells,^18^^,^^110^^,^^111^ and leads to the failure of tumor immunity. Currently, multiple EP4 receptor antagonists have advanced into clinical trials.

For instance, E7046 has undergone phase I clinical trials for the treatment of various solid tumors (NCT02540291) and as an adjuvant therapy for rectal cancer (NCT03152370). Grapiprant (Kyn Therapeutics) has been evaluated not only in a phase II clinical trial for the treatment of various solid tumors (NCT02538432) but also initiated a phase I clinical trial in combination with pembrolizumab for the treatment of colon cancer (NCT03658772), as well as phase I and II clinical trials for the treatment of non-small cell lung adenocarcinoma (NCT03696212). Furthermore, EP4 antagonist ONO-4578 has undergone phase I clinical trials in combination with nivolumab for the treatment of advanced or metastatic solid tumors (NCT03155061). Although the relationship and mechanism of action between EP4 and the tumor immune microenvironment still require further investigation, drugs targeting the antagonism of EP4 may become an important direction for clinical application in sensitizing tumor immunotherapy.

### Asthma

It is well known that PGE_2_ exhibits pro-inflammatory effects in most organs, but in the respiratory system, PGE_2_ mainly exhibits anti-inflammatory effects. The imbalance in eicosanoid metabolism (increased cysteinyl leukotrienes and reduced PGE_2_) may induce a respiratory disorder called aspirin-exacerbated respiratory disease or NSAID-exacerbated respiratory disease, which affects about 10%–20% of asthmatic patients.^112^ Although the anti-inflammatory and bronchodilatory effects of PGE_2_ in asthma are promising, they are complicated by a short half-life, low bioavailability, and undesirable side effects, including cough and airway irritation. Therefore, researchers have focused greater attention on the role of PGE_2_ receptors, especially EP2 and EP4, in the lung's immune cells to further dissect therapy for asthma.^113^^,^^114^ Asthma is the most common chronic inflammatory respiratory disease, involving a variety of inflammatory cells, including eosinophils, neutrophils, lymphoid cells, macrophages, myeloid-derived suppressor cells, regulatory T cells, and innate lymphoid cells.^115^

To date, reports on EP4 in asthma have mainly involved myeloid-derived suppressor cells and group 2 innate lymphoid cells (ILC2s). Myeloid-derived suppressor cells are innate immune cells capable of suppressing the T cell response, resulting in diminished inflammation.^116^ In an asthmatic murine model, adoptive transfer of EP4 agonist (L-902,688)-stimulated monocytic and polymorphonuclear myeloid-derived suppressor cells inhibited the production of proinflammatory cytokines (IL-4, IL-5, IL-10, IL-13, and IL-17), thus leading to a significant reduction of lung inflammation.^117^ Further studies using an aspirin-intolerant asthma model revealed that COX-1-derived PGE_2_ promoted the generation of polymorphonuclear myeloid-derived suppressor cells in the bone marrow and their recruitment to the lungs via EP4. Conversely, administration of EP4 agonists alleviated allergy-induced airway hyperresponsiveness in COX-1 knockout mice, which was associated with the development of aspirin-exacerbated respiratory disease.^118^ ILC2s are immunomodulatory cells that promote asthma, which is associated with airway hyperreactivity and inflammation, especially eosinophilic inflammation, in patients with asthma.^119^ In human and murine ILC2s, PGE_2_ has been shown to inhibit the proliferation and production of type 2 cytokines (IL-5 and IL-13) via 4-GATA binding protein 3 (GATA-3) signaling.^120^^,^^121^ Furthermore, administration of PGE_2_ or EP4 agonists (PGE1-alcohol or L-902,688) has been shown to alleviate lung pathology and ILC2 responses in IL-33-induced asthmatic mice.^121^^,^^122^ However, while Zhou et al reported that EP4-deficient mice exhibited an exacerbated inflammatory response in another ILC2-mediated asthma model induced by Alternaria extract,^121^ Robb et al demonstrated that EP2 deficiency, rather than EP4 deficiency, augmented IL-33-induced murine lung ILC2 responses *in vivo*.^122^ An interpretation for this discrepancy may be that the cAMP-elevating response of EP4 to PGE_2_ is weaker than that of EP2. Furthermore, the dominant mediator of endogenous PGE_2_ effects on the regulation of lung ILC2 responses specifically may be more dependent on EP2 than EP4. Overall, while EP2 has slightly more prominent anti-inflammatory effects than EP4 in asthma, EP4 still matters—and beyond that, it also mediates bronchodilation.^113^ The combined anti-inflammatory and bronchodilatory effects support EP4 agonists as novel therapeutics for asthma.

Finally, although EP4 is highly expressed in asthma-related inflammatory cells such as eosinophils, neutrophils, and macrophages, and *in vitro* studies in other diseases have confirmed that EP4 is involved in regulating the proliferation, adhesion, migration, invasion, and secretion of inflammatory factors in these cells, no reports have yet documented a direct association between these cells and asthma. This thus warrants further investigation.^123^

### Perspectives and prospects of targeting EP4 in the treatment of inflammatory diseases

To sum up, EP4 plays an important role in inflammatory diseases or inflammation-related diseases, and a variety of EP4 agonists or antagonists have been used in clinical trials to treat arthritis, IBD, solid tumors, and other diseases. However, in some studies, the role of EP4 in the same diseases is still controversial. On the one hand, it may be caused by the different roles of different cells of EP4 in the same disease; on the other hand, it may be caused by the diversity of downstream signaling pathways of EP4. These problems need to be clarified by more in-depth studies in a later stage. Due to the numerous adverse reactions associated with the clinical use of drugs that inhibit PGE_2_ synthesis, such as NSAIDs, EP4 mediates various physiological or pathophysiological effects of PGE_2_, and its effects are more specific. Therefore, active drugs targeting EP4 may be a new direction to replace NSAIDs in the treatment of clinical inflammatory diseases.

## CRediT authorship contribution statement

**Shuxuan Li:** Writing – original draft, Visualization, Data curation. **Tong Wang:** Writing – original draft, Visualization. **Anran Niu:** Writing – original draft, Visualization. **Yuyan Du:** Visualization, Data curation. **Dong Zhou:** Visualization, Data curation. **Liang Gao:** Data curation. **Haining Zhou:** Data curation. **Shengnan Du:** Writing – review & editing, Funding acquisition.

## Data availability

Data will be made available on request.

## Funding

This work was supported by grants from the 10.13039/501100001809National Natural Science Foundation of China (No. 81700638) and Zhengzhou University College Students' innovation and entrepreneurship training program (Henan, China) (No. 2024cxcy523, 2023CXCY371).

## Conflict of interests

The authors have no relevant financial or non-financial interests to disclose.
